# Comparison of the management of *Helicobacter pylori* infection between the older and younger European populations

**DOI:** 10.1038/s41598-023-43287-4

**Published:** 2023-10-11

**Authors:** Paulius Jonaitis, Olga P. Nyssen, Ilaria Maria Saracino, Giulia Fiorini, Dino Vaira, Ángeles Pérez-Aísa, Bojan Tepes, Manuel Castro-Fernandez, Manuel Pabón-Carrasco, Alma Keco-Huerga, Irina Voynovan, Alfredo J. Lucendo, Ángel Lanas, Samuel J. Martínez-Domínguez, Enrique Alfaro Almajano, Luis Rodrigo, Ludmila Vologzanina, Natasa Brglez Jurecic, Maja Denkovski, Luis Bujanda, Umud Mahmudov, Mārcis Leja, Frode Lerang, Gülüstan Babayeva, Dmitry S. Bordin, Antonio Gasbarrini, Juozas Kupcinskas, Oleksiy Gridnyev, Theodore Rokkas, Ricardo Marcos-Pinto, Perminder S. Phull, Sinead M. Smith, Ante Tonkić, Doron Boltin, György Miklós Buzás, Štěpán Šembera, Halis Şimşek, Tamara Matysiak-Budnik, Vladimir Milivojevic, Wojciech Marlicz, Marino Venerito, Lyudmila Boyanova, Michael Doulberis, Lisette G. Capelle, Anna Cano-Català, Leticia Moreira, Francis Mégraud, Colm O’Morain, Javier P. Gisbert, Laimas Jonaitis, Hp-EuReg investigators, Renāte Būmane, Emin Mammadov, Rustam A. Abdulkhakov, Galina Fadeenko, Jose M. Huguet

**Affiliations:** 1 Department of Gastroenterology, Lithuanian University of Health Sciences, 50161 Kaunas, Lithuania; 2 Gastroenterology Unit, Hospital Universitario de La Princesa, Instituto de Investigación Sanitaria Princesa (IIS-Princesa), Centro de Investigación Biomédica en Red de Enfermedades Hepáticas y Digestivas (CIBERehd), Universidad Autónoma de Madrid (UAM), Diego de León, 62, 28006 Madrid, Spain; 3 Department of Surgical and Medical Sciences, IRCCS AOUBO, University of Bologna, 40138 Bologna, Italy; 4 Agencia Sanitaria Costa del Sol, Red de Investigación en Servicios de Salud en Enfermedades Crónicas (REDISSEC), 29651 Marbella, Spain; 5 Department of Gastroenterology, AM DC Rogaska, 3250 Rogaska Slatina, Slovenia; 6 Department of Gastroenterology, Hospital de Valme, 41014 Seville, Spain; 7 Department of Gastroenterology, A.S. Loginov Moscow Clinical Scientific Center, 111123 Moscow, Russia; 8 Department of Gastroenterology, Hospital General de Tomelloso, 13700 Tomelloso, Spain; 9 IIS Aragón y Facultad de Medicina de la Universidad de Zaragoza, 50009 Zaragoza, Spain; 10 Gastroenterology Unit, Hospital Universitario Central de Asturias, 33011 Oviedo, Spain; 11 Department of Gastroenterology, Gastrocentre, 614068 Perm, Russia; 12 Department of Gastroenterology, Interni Oddelek, Diagnostic Centre, 4260 Bled, Slovenia; 13 Hospital Donostia, Instituto Biodonostia, Centro de Investigación Biomédica en Red de Enfermedades Hepáticas y Digestivas (CIBERehd), Universidad del País Vasco (UPV/EHU), 20018 San Sebastián, Spain; 14 Modern Hospital, 1119 Baku, Azerbaijan; 15 Department of Gastroenterology, Digestive Diseases Centre Gastro, Institute of Clinical and Preventive Medicine and Faculty of Medicine, University of Latvia, Riga, 1079 Latvia; 16 Department of Gastroenterology, Østfold Hospital Trust, 1714 Grålum, Norway; 17 Memorial Klinik, 1096 Baku, Azerbaijan; 18 Department of Gastroenterology, A.S. Loginov Moscow Clinical Scientific Center, 111123 Moscow, Russia; 19 A.I. Yevdokimov Moscow State University of Medicine and Dentistry, 127473 Moscow, Russia; 20 Tver State Medical University, 170100 Tver, Russia; 21 Medicina Interna, Fondazione Policlinico Universitario A. Gemelli Istituto di Ricovero e Cura a Carattere Scientifico, Università Cattolica del Sacro Cuore, 00168 Rome, Italy; 22 Government Institution “L.T.Malaya Therapy National Institute of the National Academy of Medical Sciences of Ukraine”, Kyiv, Ukraine; 23 Department of Gastroenterology, Henry Dunant Hospital, 115 26 Athens, Greece; 24 Department of Gastroenterology, Centro Hospitalar do Porto Institute of Biomedical Sciences Abel Salazar, Centro de Investigação em Tecnologias e Serviços de Saúde, University of Porto, 4050-313 Porto, Portugal; 25 Department of Gastroenterology, Aberdeen Royal Infirmary, Aberdeen, AB25 2ZN UK; 26 Faculty of Health Sciences, Trinity College Dublin, Dublin, D02PN40 Ireland; 27 Department of Gastroenterology, University Hospital of Split, University of Split School of Medicine, 21000 Split, Croatia; 28 Division of Gastroenterology, Rabin Medical Center, Sackler School of Medicine, Tel Aviv University, 49100 Tel Aviv, Israel; 29 Department of Gastroenterology, Ferencváros Health Centre, 1095 Budapest, Hungary; 30 2nd Department of Internal Medicine and Gastroenterology, University Hospital and Charles University, Faculty of Medicine in Hradec Králové, 500 03 Hradec Králové, Czech Republic; 31 Division of Gastroenterology and Hepatology, Hacettepe University School of Medicine, 06230 Ankara, Turkey; 32 Department of Gastroenterology, CHRU de Nantes, Hôpital Hôtel Dieu, 44000 Nantes, France; 33 Department of Gastroenterology, Clinical Center of Serbia, University of Belgrade School of Medicine, 11000 Belgrade, Serbia; 34 Department of Gastroenterology, Pomeranian Medical University, 70-204 Szczecin, Poland; 35 Department of Gastroenterology, Otto-Von-Guericke University, 39120 Magdeburg, Germany; 36 Department of Gastroenterology, Medical Microbiology, Medical University of Sofia, 1431 Sofia, Bulgaria; 37 Division of Gastroenterology and Hepatology, Medical University Department, Kantonsspital Aarau, 5001 Aarau, Switzerland; 38 Department of Gastroenterology, Meander Medical Center, 3813 TZ Amersfoort, The Netherlands; 39 GOES Research Group, Althaia Xarxa Assistencial Universitària de Manresa, 08243 Manresa, Spain; 40 Department of Gastroenterology, Hospital Clínic Barcelona, Centro de Investigación Biomédica en Red en Enfermedades Hepáticas y Digestivas (CIBEREHD), IDIBAPS (Institut d’Investigacions Biomèdiques August Pi i Sunyer), University of Barcelona, 08036 Barcelona, Spain; 41 INSERM U1312, Université de Bordeaux, 33000 Bordeaux, France; 42 Internal Medicine and Gastroenterology Department, Azerbaijan State Advanced Training Institute for Doctors named after A. Aliyev, Baku, Azerbaijan; 43 Kazan State Medical University, Kazan, Tatarstan, Russia; 44 Digestive Ukrainian Academy of Medical Sciences, Kyiv, Ukraine; 45 Gastroenterology Unit, Consorci Hospital General Universitari Valencia, Valencia, Spain

**Keywords:** Gastrointestinal diseases, Infectious diseases, Geriatrics, Therapeutics, Diseases, Gastroenterology, Health care, Signs and symptoms, Medical research, Epidemiology, Antibiotics, Infectious-disease epidemiology, Microbiology, Infectious-disease diagnostics

## Abstract

The prevalence of *Helicobacter pylori* remains high in the older population. Specific age-related peculiarities may impact the outcomes of *H*. *pylori* treatment. The aim of the study was to evaluate the diagnostics and effectiveness of *H*. *pylori* eradication between the younger and older European populations. “European Registry on *H. pylori* Management (Hp-EuReg)” data from 2013 to 2022 were analyzed. Patients were divided into older (≥ 60 years) and younger (18–59 years) groups. Modified intention-to-treat (mITT) and per-protocol (PP) analysis was performed. 49,461 patients included of which 14,467 (29%) were older-aged. Concomitant medications and penicillin allergy were more frequent among the older patients. Differences between younger and older populations were observed in treatment duration in first-line treatment and in proton pump inhibitors (PPIs) doses in second-line treatment. The overall incidence of adverse events was lower in the older adults group. The overall first-line treatment mITT effectiveness was 88% in younger and 90% in the older patients (p < 0.05). The overall second-line mITT treatment effectiveness was 84% in both groups. The effectiveness of the most frequent first- and second-line triple therapies was suboptimal (< 90%) in both groups. Optimal efficacy (≥ 90%) was achieved by using bismuth and non-bismuth-based quadruple therapies. In conclusion, the approach to the diagnostics and treatment of *H*. *pylori* infection did not generally differ between younger and older patients. Main differences were reported in the concurrent medications, allergy to penicillin and adverse events both in first- and second-line treatment. Optimal effectiveness rates were mostly achieved by using bismuth and non-bismuth-based quadruple therapies. No clinically relevant differences in the effectiveness between the age groups were observed.

## Introduction

*Helicobacter pylori* (*H. pylori*) is the main cause of chronic gastritis as well as one of the main etiopathogenetic factors in the development of peptic ulcer disease. It is the only bacterium that is classified as Class I (definite) carcinogen by World Health Organization (WHO) and is the main risk factor in the etiopathogenesis of gastric adenocarcinoma and mucosa-associated lymphoid tissue lymphoma^1–4^.

Despite the fact that the prevalence of *H. pylori* is declining, especially in the younger population^5,6^, it is estimated that around 50% of the world’s populations is still infected with this bacterium^7^. Based on a wide systematic review and meta-analysis, the prevalence of *H. pylori* in Western Europe is 34%^8^ and is significantly higher in Eastern and Southern European countries^9^.

The prevalence of *H. pylori* is related to the cohort effect, meaning that it is higher among the cohorts with earlier date of birth, i.e., the older the cohort, the higher the prevalence of *H. pylori*. The vast majority of people acquire this bacterium in their early childhood (up to 10 years of age) and the possibility of a later infection is rather low^10^. After the treatment of *H. pylori* the reinfection rate is also low^11^; however it might be higher in the areas with low socioeconomic status and high prevalence of *H. pylori*.

Epidemiological studies have reported that the world’s population is shifting towards older adults (> 60 years old) and the number of persons aged over 60 years is expected to double in the upcoming 10 years^12,13^. It has been reported that the prevalence of *H. pylori* in older population is significantly higher compared to younger people^14^, resulting in higher incidence of gastric cancer, peptic ulcer disease and other *H. pylori*-associated conditions in this population^15^. Even though there is a clear lack of epidemiological research in older patients, the available studies have reported the prevalence of *H. pylori* ranging from 40% up to 75% in these subjects^14,15^. Older adults are also associated with other age-related peculiarities, such as a higher number of comorbidities and concurrent medications, lower treatment compliance, impaired renal function, changes in drug metabolism^16,17^ as well as other physiological changes^18^.

Both the currently updated Maastricht VI/Florence, as well as previous Maastricht V/Florence, Consensus Reports^4,19^ on the management of *H. pylori* infection have decided not to separate the recommended treatment regimens in different age groups as it has been reported that these regimens are equally effective both in older and younger patients^20^. In fact, the limited available studies have reported that the standard triple therapy achieved close to optimal or, in seldom cases, optimal effectiveness and most of the quadruple therapies and sequential therapies have been reported to achieve optimal (> 90%) eradication rates in older-aged groups^19–21^. It has been described that *H. pylori* eradication prescriptions are safe and effective in older adults, although the experience is quite confined^21,22^.

There is a scarcity of data not only regarding the *H. pylori* diagnostics and treatment in older patients but also comparing the results with younger subjects. The latter could evaluate more in depth both population’s characteristics and potentially help in the therapeutical tailoring in the different age groups. In fact, after performing a literature search, we could not retrieve any European studies in recent years that have compared the diagnostics and treatment of *H. pylori* between older and younger populations.

On the other hand, it is known that *H. pylori* resistance rates to antibiotics are increasing worldwide. An extensive analysis reported that primary clarithromycin resistance in European countries has doubled in the last 20 years^23^. It is likely that older adults might have patterns of higher antimicrobial resistance due to the lifelong exposure to various antibiotics^24,25^. However, a population based study in China revealed that the failure rates of clarithromycin containing triple therapy were especially high in younger populations, whereas the higher cure rates were observed in the older subjects^26^.

The aim of this study was therefore to evaluate and compare the diagnostic methods and treatment prescriptions for *H. pylori* infection as well as the effectiveness of the most frequent first- and second-line *H. pylori* eradication regimens between the older and younger adults in Europe.

## Methods

### European registry on *H. pylori* management (Hp-EuReg)

The Hp-EuReg is an international, multicentre, prospective, non-interventional registry that has been recording information on the management of *H. pylori* infection since the year 2013. The Hp-EuReg protocol^27^ establishes national coordinators in the selected 29 countries, where gastroenterologists have been recruited at over 300 study centres to provide input to the registry.

### Participants and study groups

This study data was obtained from the centres of all the participating countries. The participants were patients that were included in the Hp-EuReg since the year 2013 up to the January of 2022. All of the patients were adults, who were diagnosed with *H. pylori* infection.

The study participants were divided into two groups based on the age: younger (18–59 years old) and older (≥ 60 years old). The age cut-off value between the groups was selected based on the “older adults” definition by WHO and some sources from the United Nations (UN)^28,29^.

### Data collection

Data were collected through an electronic Case Report Form (e-CRF), collecting the patient’s demographic information, any previous eradication attempts, and the treatments employed, as well as the outcomes of the treatment, recording details such as the compliance, the cure rate, the follow-up, etc. and the adverse events (AEs). This information was registered at the REDCap database^30^ managed and hosted by the "Asociación Española de Gastroenterología" (AEG; http://www.aegastro.es), a non-profit Scientific and Medical Society that focuses on Gastroenterology research.

### Data management

Data were quality reviewed by evaluating whether the study selection criteria had been met, whether information was correctly registered and ultimately to ensure the study was conducted according to the highest scientific and ethical standards, in accordance with the latest revision of the ethical guidelines firstly announced in 1975 Declaration of Helsinki. Data discordances were resolved by querying the investigators and through group emailing.

### Variables categorization and definitions

PPI doses were categorized according to the potency of acid inhibition, as low-dose (4.5–27 mg of omeprazole equivalents given twice a day), standard-dose (32–40 mg of omeprazole equivalents given twice a day), or high-dose (54–128 mg of omeprazole equivalents given twice a day)^31,32^. Likewise, the duration of treatment was categorized as 7, 10, or 14 days to ease the interpretation. Sub-analyses were performed according to the treatment duration and PPI doses comparing both age groups (younger and older subjects).

The following categories were used for the most frequently prescribed first-line treatments: Triple-CA/M (clarithromycin, amoxicillin/metronidazole), Seq CAT-CAM (clarithromycin, amoxicillin, tinidazole—clarithromycin, amoxicillin, metronidazole), Quad-CAT-CAM, Quad-Pylera^®^ (three-in-one single-capsule containing metronidazole, tetracycline and bismuth) + Quad Bi (bismuth), Quad-CAB (clarithromycin, amoxicillin, bismuth) and other. Likewise, following categories were used for the most frequent second-line treatments: Quad Pylera^®^ + Quad Bi, Quad-LAB (levofloxacin, amoxicillin, bismuth), Triple-AL (amoxicillin, levofloxacin), Conco-Seq (concomitant-sequential), and other.

In order to calculate the effectiveness of different *H. pylori* eradication regimens, per-protocol (PP), intention to treat (ITT) and modified intention-to-treat (mITT) analyses were used. The ITT analysis includes all cases registered in Hp-EuReg, allowing at least a 6-month follow-up, and lost to follow-up cases were considered treatment failures. The PP analysis includes all cases that have completed follow-up (i.e., had undertaken a valid confirmatory test after the eradication treatment) and had taken at least 90% of the treatment drugs, as defined in the protocol. The mITT was designed to reach the closest results to those obtained in clinical practice, and therefore included all cases that had completed the follow-up, regardless of the compliance to treatment. All the patients, that were empirically treated (that is, without prescribing a susceptibility-guided antibiotic treatment) were included in the effectiveness analysis.

AEs and compliance were evaluated through patient questioning with both open-ended questions and a predefined questionnaire, by face-to-face interview. Compliance was defined, through physician questioning, as having taken at least 90% of the prescribed drugs.

AEs were classified depending on the intensity of symptoms evaluated by the corresponding physician: mild (not interfering with daily routine), moderate (affecting daily routine), intense/severe (not allowing normal daily routine), and serious (causing death, hospitalization, disability, congenital anomaly, and/or requiring intervention to prevent permanent damage).

### Statistical analyses

Continuous variables were summarized as the mean and standard deviation, while qualitative variables were presented as the absolute relative frequencies, displayed as percentages (%) together with their 95% CIs, where applicable. The selected level of statistical significance was p < 0.05 (two-tailed).

All treatments were accounted in the descriptive and univariate analyses; for the purpose of the multivariate analysis, logistic regression (LR) was performed first controlling by the most frequently prescribed first-line treatments and then by the most frequently second-line treatments.

The LR used a backward modelling strategy and compared models using the log-likelihood ratio. The mITT population was the dependent variable used to evaluate the association between the treatments' eradication rate and the following independent variables: age [ref. 18–59 years old group], sex [ref. female], indication [ref. dyspepsia], compliance [ref. No, < 90% drug intake], PPI dose [ref. low dose], treatment length [ref. 7 days] and prescribed eradication regimens (for the first line-treatment [ref. Triple-CA/M]; for the second-line treatment [ref. Quad Pylera^®^ + Quad Bi] as defined earlier]. In the multivariate analysis, the effect was evaluated by calculating odds ratios (ORs) and the corresponding 95% confidence intervals (95% CIs).

### Ethics approval statement

The study was approved by the Ethics Committee of La Princesa University Hospital (Madrid, Spain). It was registered at ClinicalTrials.gov (NCT02328131).

## Results

### Baseline characteristics

**Figure 1:**
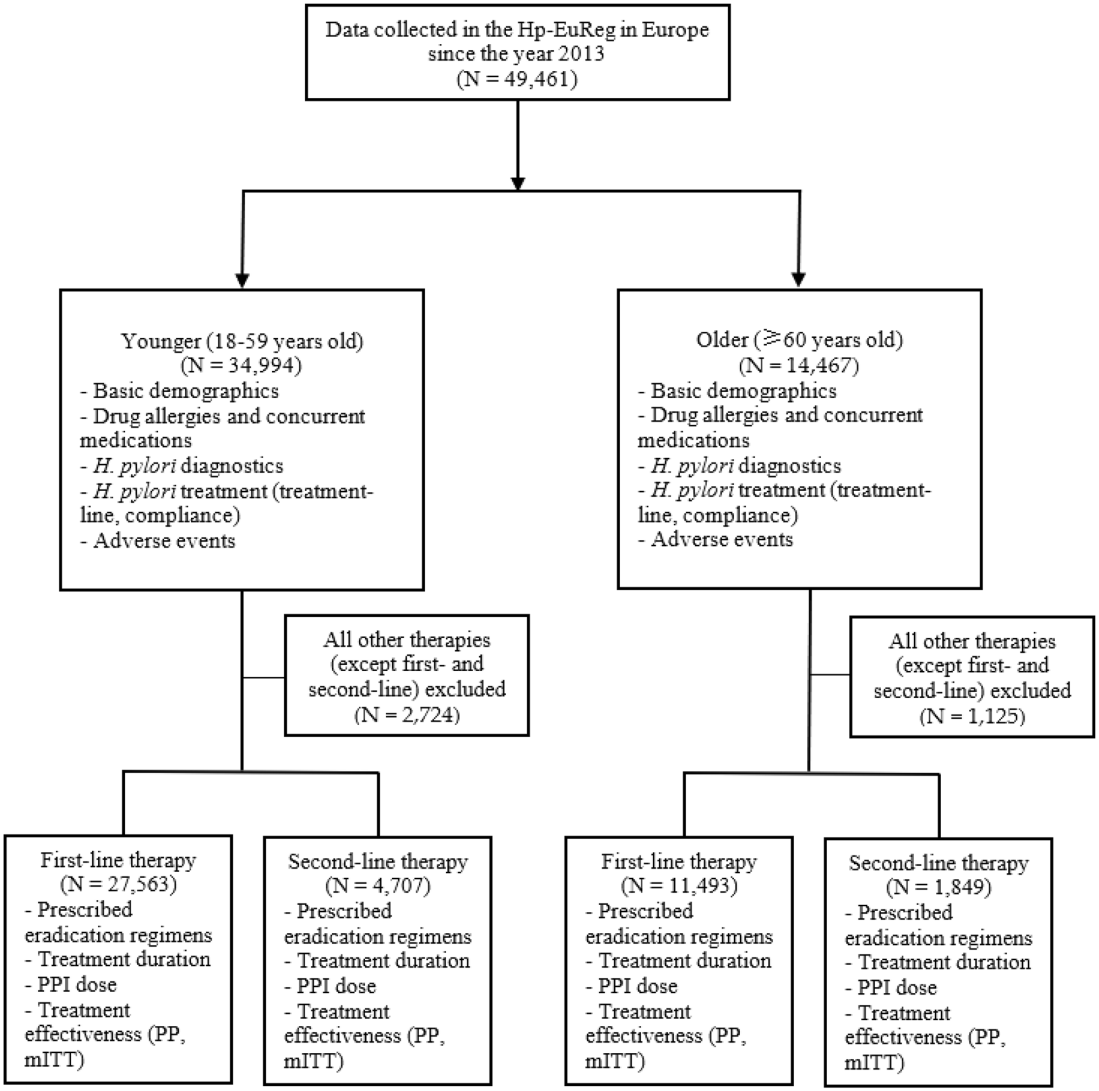
Study flow chart. *PP* per-protocol, *mITT* modified Intention-to-treat.

Figure 1 depicts the flow-chart of the study. The main results of the differences in the demographics, diagnostics, and treatment of *H. pylori* infection between the older and younger age groups is presented in Table 1.

**Table 1:** Demographics, diagnostics, and treatment prescriptions in *H. pylori* management between older and younger European populations.

	Older (≥ 60 years)	Younger (18–59 years)	p-value
**Total number of patients, N (%)**	14,467 (29%)	34,994 (71%)	
**Gender, N (%)**			
Male	5365 (37%)	14,244 (41%)	** < 0.05**
Female	9093 (63%)	20,730 (59%)	** < 0.05**
**Average age (mean ± standard deviation)**	68.1 ± 6.3	42.8 ± 10.7	** < 0.05**
**Main treatment indications, N (%)**			
Uninvestigated dyspepsia	5111 (36%)	13,248 (38%)	** < 0.05**
Peptic ulcer disease	2893 (20%)	5094 (15%)	** < 0.05**
**Main symptoms, N (%)**			
Dyspepsia	10,571 (73%)	27,070 (77%)	** < 0.05**
Heartburn	3831 (26.3%)	9183 (26.5%)	> 0.05
**Concurrent medications, N (%)**	7364 (55%)	8834 (27%)	** < 0.05**
**Drug allergies, N (%)**	796 (6%)	1306 (4%)	** < 0.05**
Penicillin	650 (4.5%)	1050 (3%)	** < 0.05**
Macrolides	43 (0.3%)	92 (0.3%)	> 0.05
Fluoroquinolones	27 (0.2%)	50 (0.1%)	> 0.05
**Main diagnostic methods pre-treatment, N (%)**			
Histology	6840 (47%)	13,191 (38%)	** < 0.05**
Rapid urease test	5463 (38%)	13,138 (38%)	> 0.05
Urea breath test	3550 (25%)	10,702 (31%)	** < 0.05**
**Main diagnostic tests post-treatment, N (%)**			
Urea breath test	9544 (66%)	21,755 (62%)	** < 0.05**
Stool antigen test	1968 (13.6%)	5401 (15.4%)	** < 0.05**
Histology	715 (5%)	1529 (4%)	** < 0.05**
**Treatment compliance, N (%)**	12,938 (97%)	31,270 (97%)	> 0.05
**First-line prescriptions, N (%)**			
Triple-therapy	4851 (42%)	12,492 (45%)	** < 0.05**
Quadruple-therapy	5182 (45%)	12,641 (46%)	** < 0.05**
**Second-line prescriptions, N (%)**			
Triple-therapy	788 (43%)	2134 (45%)	** < 0.05**
Quadruple-therapy	956 (52%)	2393 (51%)	** < 0.05**
**PPI potency in first-line treatment, N (%)**			
Low	5275 (46%)	12,312 (45%)	> 0.05
Standard	2726 (24%)	6831 (25%)	> 0.05
High	3516 (31%)	8539 (31%)	> 0.05
**PPI potency in second-line treatment, N (%)**			
Low	750 (40%)	1691 (36%)	** < 0.05**
Standard	423 (23%)	1059 (23%)	> 0.05
High	705 (38%)	1983 (42%)	** < 0.05**
**First-line treatment duration, N (%)**			
7 days	1583 (14%)	3170 (12%)	** < 0.05**
10 days	5700 (50%)	13,351 (49%)	** < 0.05**
14 days	4185 (37%)	11,019 (40%)	** < 0.05**
**Second-line treatment duration, N (%)**			
7 days	75 (4%)	197 (4%)	> 0.05
10 days	1023 (56%)	2618 (56%)	> 0.05
14 days	744 (40%)	1881 (40%)	> 0.05
**Adverse events**			
Overall, N (%)	3062 (23%)	8144 (25%)	** < 0.05**
Treatment cessation due to AEs, %	1.5%	1.2%	> 0.05

*PPI*—proton pump inhibitor.

Significant p-values are in bold.

Since the year 2013, data from 49,461 cases were included in the Hp-EuReg and used for current analysis. As already mentioned, the patients were divided into two groups based on their age: there were 14,467 (29%) older (aged 60 years or older) and 34,994 (71%) younger (18–59 years old) patients. There were 63% female in the older group and 59% female in the younger group (p < 0.05 between the age groups).

The older adults group reported a significantly higher intake of concurrent medications (the main evaluated drug groups were PPIs, acetylsalicylic acid, non-steroidal anti-inflammatory drugs and statins) as compared to the younger subjects (55% vs. 27% respectively, p < 0.05). Older-aged group was characterized by statistically significantly higher rate of allergy to penicillin (5% in the older compared to 3% in the younger group, p < 0.05), whereas there were no differences in other evaluated drug groups, such as macrolides (0.3% in both groups) and fluoroquinolones (0.2% vs. 0.1% respectively, p > 0.05).

Before *H. pylori* eradication dyspeptic symptoms were more frequently reported in older subjects as compared to the younger ones (77% vs. 73% respectively, p < 0.05), whereas there were no differences in the prevalence of heartburn (both groups 26%, p > 0.05). The main indications for the treatment of *H. pylori* infection in the older and younger age groups were dyspeptic syndrome (36% vs. 38% respectively) and peptic ulcer disease (20% vs. 15% respectively, p < 0.05). Further causes (for instance, gastroesophageal reflux disease, erosive gastritis etc.) accounted for 32% of the indications in older and 29% in younger patients groups (p < 0.05).

The main *H. pylori* diagnostic tests used in older and younger subjects before the treatment were histological examination (47% and 38% respectively, p < 0.05), rapid urease test (RUT) (both groups 38%, p > 0.05), urea breath test (UBT) (25% and 31% respectively, p < 0.05), microbiological culture (11% and 10% respectively, p < 0.05) and serology (7% vs. 8% respectively, p < 0.05). The main diagnostic tests post-treatment in both groups were UBT (66% and 62% respectively, p < 0.05), stool antigen test (14% and 15% respectively, p < 0.05), histological examination (5% and 4% respectively, p < 0.05) and RUT (3% in both groups, p > 0.05).

### Prescriptions, treatment duration and PPIs

In both age groups 81% of the cases were treatment-naive. In the first-line treatment, quadruple therapy was prescribed in 45% of the older and 46% of the younger subjects (p < 0.05) and triple therapy in 42% and 45% respectively (p < 0.05). In the second-line treatment quadruple therapy was prescribed in 52% of the older and 51% of the younger age cases (p < 0.05) and triple therapy in 43% and 45% respectively (p < 0.05).

The overall duration of first-line treatment in older and younger adults was 7 days in 14% and 12% of the cases respectively; 10 days in 50% and 49% respectively; and 14 days in 36% and 40% respectively (p < 0.05 in all groups). The overall duration of the second-line treatment was as follows: 7 days in 4%, 10 days in 56% and 14 days in 40% of the cases in both age groups (p > 0.05).

In the first-line treatment low PPI doses were most frequently prescribed in both groups and no significant differences in prescribed PPI doses were found between the groups. In the second-line treatment low doses of PPIs were most frequently prescribed in older adults (40% vs 36% respectively, p < 0.05) and high doses of PPIs most frequently in the younger adults (38% vs 42% respectively, p < 0.05).

### Treatment effectiveness

**Figure 2:**
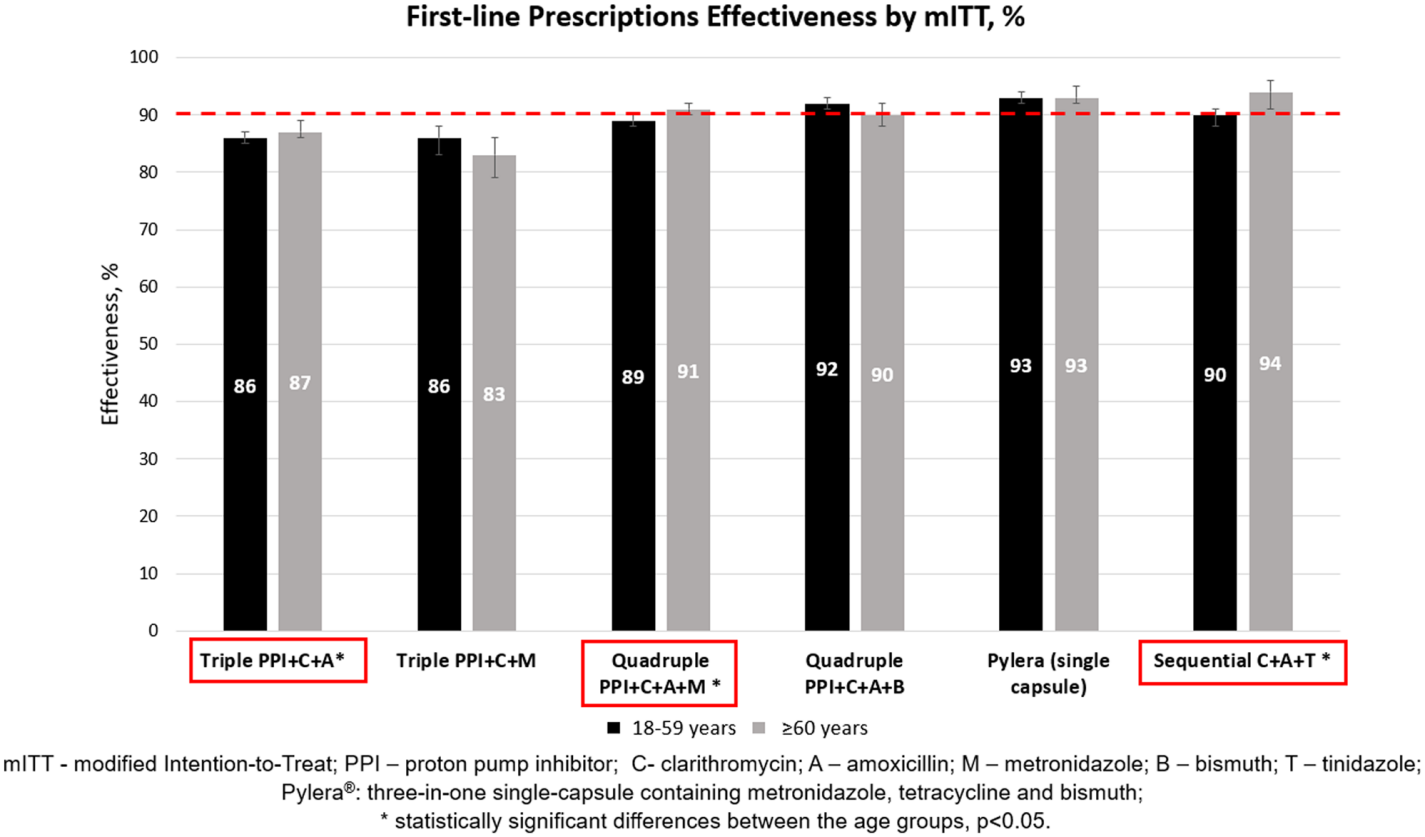
Comparison of the mITT effectiveness of 6 most frequent first-line prescriptions between the younger and older adults.

**Figure 3:**
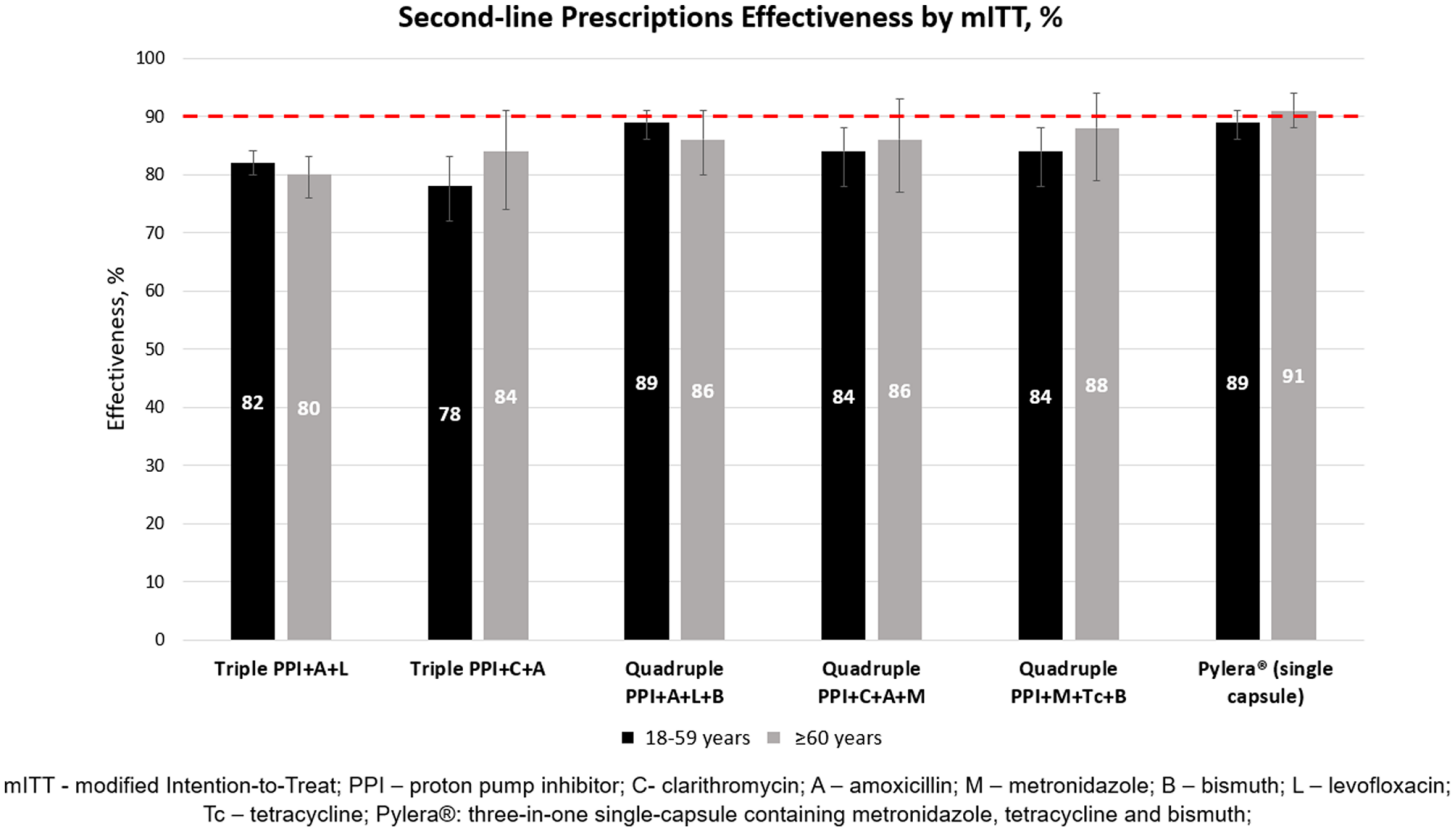
Comparison of the mITT effectiveness of six most frequent second-line prescriptions between the younger and older adults.

The detailed analysis of the most widely used first- and second-line prescriptions in the older and younger age groups and their effectiveness (ITT, mITT and PP) comparison is presented in Table 2. The graphic comparison of the mITT effectiveness of the six most frequently prescribed first- and second-line treatments between the age groups is illustrated in Figs. 2 and 3, respectively.

**Table 2:** First- and second-line *H. pylori* treatment effectiveness in the younger and older European populations.

Treatment	Younger adults (18–59 years)				Older adults (≥ 60 years)			
	Use, N	PP % (95% CI)	mITT % (95% CI)	ITT % (95% CI)	Use, N	PP % (95% CI)	mITT % (95% CI)	ITT % (95% CI)
**Effectiveness of first-line treatment prescriptions between the younger and older populations**								
Triple PPI + C + A	10,540	87 (86–87)	86 (85–87)*	67 (66–68)*	4065	88 (87–89)	87 (86–89)*	71 (69–72)*
Triple PPI + C + M	1150	86 (84–88)	86 (83–88)	68 (65–71)	491	83 (79–86)	83 (79–86)	71 (66–75)
Triple PPI + A + L	425	83 (79–87)	83 (78–86)	76 (72–80)	162	83 (75–88)	83 (76–89)	76 (69–82)
Quadruple PPI + C + A + M	4199	90 (89–91)*	89 (88–90)*	86 (85–87)*	1940	91 (90–93)*	91 (90–92)*	89 (87–90)*
Quadruple PPI + C + A + B	3489	92 (91–93)	92 (91–93)	76 (75–77)	1086	91 (89–93)	90 (88–92)	76 (73–79)
Quadruple PPI + C + A + T	309	98 (95–99)	96 (93–98)	92 (88–95)	127	96 (89–99)	92 (85–96)	86 (78–92)
Pylera^®^ (single capsule)^1^	3233	94 (93–95)	93 (92–94)	89 (87–90)	1519	94 (93–95)	93 (92–95)	89 (87–91)
Sequential C + A + T	1291	90 (88–92)*	90 (88–91)*	80 (78–82)*	623	94 (92–96)*	94 (91–96)*	85 (81–87)*
Sequential C + A + M	458	84 (80–87)	82 (78–86)	76 (72–80)	222	84 (78–89)	82 (76–87)	75 (69–81)
**Effectiveness of second-line treatment prescriptions between the younger and older populations**								
Triple PPI + A + L	1422	83 (80–85)	82 (80–84)	74 (71–76)	524	80 (76–84)	80 (76–83)	73 (69–77)
Triple PPI + C + A	323	78 (72–83)	78 (72–83)	60 (54–65)	102	84 (74–91)	84 (74–91)	68 (58–77)
Triple PPI + A + R	105	80 (70–87)	79 (69–87)	68 (58–76)	49	82 (67–92)	82 (67–92)	74 (59–85)
Triple PPI + A + Mx	105	92 (85–97)	92 (85–97)	90 (82–95)	35	87 (70–96)	87 (70–96)	77 (60–90)
Quadruple PPI + A + L + B	584	89 (86–92)	89 (86–91)	73 (69–76)	225	86 (80–91)	86 (80–91)	75 (69–81)
Quadruple PPI + C + A + M	234	84 (79–89)	84 (78–88)	80 (74–85)	91	86 (77–92)	86 (77–93)	83 (73–90)
Quadruple PPI + M + Tc + B	224	86 (80–91)	84 (78–88)	75 (69–81)	101	87 (78–93)	88 (79–94)	77 (68–85)
Quadruple PPI + C + A + B	229	91 (85–96)*	91 (85–95)*	52 (45–59)	66	79 (64–89)*	80 (66–90)*	60 (47–72)
Pylera^®^ (single capsule)^1^	843	89 (87–91)	89 (86–91)	83 (80–86)*	384	92 (89–95)	91 (88–94)	88 (84–91)*

*PP*—per protocol, *mITT*—modified Intention-To-Treat, *ITT*—Intention-To-Treat, *95% CI*—95% confidence interval, *PPI*—proton pump inhibitor, *C*—clarithromycin, *A*—amoxicillin, *M*—metronidazole, *B*—bismuth, *T*—tinidazole, *L*—levofloxacin, *Tc*—tetracycline, *Mx*—moxifloxacin, *R*—rifabutin.

^1^Pylera^®^: three-in-one single-capsule containing metronidazole, tetracycline and bismuth.

*Statistically significant differences between the age groups, p < 0.05.

The overall first-line treatment effectiveness was 89% (95% CI 89–90%) by PP and 88% (95% CI 88–89%) by mITT in the younger and 89% (95% CI 88–89%) by PP and 90% (95% CI 89–91%) by mITT in the older groups. The effectiveness of most frequently prescribed first-line regimen, which was standard triple therapy (PPI + C + A), was 88% by PP and 87% by mITT in the older group, whereas it was 87% by PP and 86% by mITT in the younger group (p < 0.05 only comparing the mITT effectiveness), meaning it reached suboptimal (< 90%) efficacy rates in either group. The remaining triple therapies failed to reach optimal eradication rates as well. Optimal (≥ 90%) eradication rates in the first-line treatment were achieved by using quadruple therapies (quadruple PPI + C + A + B, quadruple PPI + C + A + T and single-capsule bismuth quadruple therapy) and the most frequently used sequential therapy (PPI + C + A + T) in both age groups. Statistically significant differences in the eradication effectiveness between the age groups were reported when standard triple therapy (PPI + C + A), quadruple PPI + C + A + M or sequential PPI + C + A + T therapies were used.

The overall second-line treatment effectiveness was 84% both by PP and by mITT in the younger and older subjects. The efficacy of the most popular second-line prescription PPI + A + L was 80% by PP and by mITT in the older adults group, whereas it was 83% by PP and 82% by mITT in the younger adults group (p > 0.05 by both analyses), meaning that it also achieved only suboptimal eradication rates. Optimal eradication rates in the younger group were achieved by using triple PPI + A + Mx (moxifloxacin) or quadruple PPI + C + A + B therapies and in the older group only single-capsule bismuth quadruple therapy managed to reach optimal treatment effectiveness. Statistically significant difference between the age groups in the second-line treatment effectiveness was only reported when quadruple PPI + C + A + B therapy was used.

### Multivariate analysis

The detailed multivariate analysis of the first- and second-line treatments is presented in Table 3. After performing the multivariate analysis it has been calculated that compliance with treatment, quadruple bismuth-based prescriptions (including single-capsule bismuth quadruple therapy), longer treatment durations (14 days vs. 7 days; 10 days vs. 7 days), higher acid inhibition (high PPIs doses vs. low; medium PPIs doses vs. low), sequential CAT/CAM, quadruple CAT/CAM and quadruple CAB prescriptions as well as belonging to older adults group were the main factors associated with higher mITT cure rate in the first-line treatment.

**Table 3:** Independent variables associated with higher mITT rates in the first- and second-line treatments.

Variable	OR	95% CI	p-value
**First-line treatment**			
Treatment compliance	7.34	6.12–8.80	< 0.005
Quadruple bismuth-based prescriptions	2.36	2.06–2.70	< 0.005
14 days vs. 7 days treatment duration	1.73	1.54–1.95	< 0.005
High PPIs doses vs. low PPIs doses	1.64	1.48–1.80	< 0.005
Sequential CAT/CAM prescriptions	1.52	1.30–1.77	< 0.005
Medium PPIs doses vs. low PPIs doses	1.42	1.27–1.57	< 0.005
Quadruple CAT/CAM prescriptions	1.29	1.16–1.43	< 0.005
10 days vs. 7 days treatment duration	1.17	1.05–1.31	< 0.005
Quadruple CAB prescription	1.17	1.02–1.35	< 0.05
Older adults group	1.13	1.05–1.12	< 0.005
**Second-line treatment**			
14 days vs. 7 days treatment duration	4.45	3.21–6.17	< 0.005
10 days vs. 7 days treatment duration	3.76	2.80–5.04	< 0.005
Treatment compliance	3.56	2.38–5.23	< 0.005
High PPIs doses vs. low PPIs doses	2.17	1.80–2.61	< 0.005
Medium PPIs doses vs. low PPIs doses	1.52	1.25–1.83	< 0.005

*OR*—odds ratio, *95% CI*—95% confidence interval, *PPIs*—proton pump inhibitors, *CAT*—clarithromycin, amoxicillin, tinidazole, *CAM*—clarithromycin, amoxicillin, metronidazole, *CAB*—clarithromycin, amoxicillin, bismuth salts, *mITT*—modified intention-to-treat.

In the second-line treatment the main factors associated with higher mITT rates were longer treatment durations (14 days vs. 7 days; 10 days treatment vs. 7 days), good treatment compliance and higher PPIs doses (high PPIs doses vs. low; medium PPIs doses vs. low). Compared to quadruple bismuth-based prescriptions (including single-capsule bismuth quadruple therapy), Triple-AL and Conco-Seq prescriptions were associated with significantly lower mITT rates.

### Compliance and safety

The treatment compliance was 97% in both groups. The overall AEs rate was lower in older subjects compared to the younger adults (23%; 95% CI 24–26% vs. 25%; 95% CI 22–24% respectively, p < 0.05), however severe AEs were more frequent in older patients. The most frequent AEs in older and younger subjects were dysgeusia (7% in both groups), nausea (7% and 8% respectively), diarrhea (6% and 8% respectively) and vomiting (2% and 3% respectively). Most of the AEs were mild to moderate in intensity and lasted 7–14 days. The most frequent severe AEs among the older and younger age groups were asthenia (23% and 11% respectively, p < 0.05), anorexia (16% and 10% respectively, p < 0,05) and abdominal pain (8% and 7% respectively, p > 0.05). Among the older adults 1.5% had to stop taking medications due to AEs, whereas it was 1.2% in the younger group (p > 0.05).

## Discussion

In this study we have evaluated the differences of *H. pylori* diagnostics and treatment between the older and younger European populations. We would like to point out that such analysis is one of only very few available both evaluating an older-aged population and comparing it to younger subjects. Additionally, this is one of only few studies providing data on such a large number of patients from all Europe and enabling more accurate data and more reliable statistical results.

Our analysis confirmed the expected epidemiological hypothesis, regarding the baseline characteristics—compared to the younger patients; more older subjects were taking concurrent medications and reported a higher rate of allergy to penicillin. Even though a higher number of concurrent medications and higher rate of drug allergies is usually associated with worse treatment compliance^33,34^, this was not confirmed in our study and had no influence on the efficacy of the eradication therapy.

The diagnostics of *H. pylori* also complied with the current guidelines^4,19^ and the study confirmed that the invasive diagnostic methods (histology, RUT) were more frequent for the initial diagnosis of *H. pylori* prior to the treatment and, as anticipated, some of these methods were also more common in the older population. For the confirmation of eradication UBT was the preferred choice, followed by SAT in both age groups.

Most of the performed studies^35–37^ have revealed that quadruple therapies were superior to triple therapies regarding the effectiveness and, in fact, our study has demonstrated that quadruple therapies were more frequently prescribed for the first- and second-line treatment, in both age groups. The most frequent treatment duration was 10–14 days in both age groups, in line with the current guidelines. Seven days duration treatment is no longer recommended^4,37,38^ and even though there were some cases with 7 days treatment duration in both age groups, these cases were registered in the early years of the registry, as reported in previous Hp-EuReg research and, also in accordance with the Maastricht V/Florence consensus report, updated in the year 2016, where 7 days treatment duration was no longer recommended.

We could have expected that the doses of PPI might have been lower in the older population due to the higher chance of possible AEs (e.g., diarrhea, *Clostridioides difficile* infection); however we did not find any significant differences between the age groups regarding the PPI dose in the first line therapy and the differences in the second-line were not clinically relevant. The multivariate analysis revealed that high doses of PPIs were associated with better mITT rate in the first- and second-line therapies; however, low doses of PPIs were prescribed most frequently in the first-line treatment in both age groups. We could speculate that some of the prescribing gastroenterologists were not acquainted with the guidelines or were cautious of the possible AEs, especially in older patients. On the other hand, we should also point out that there was a clear shift from low doses of PPIs to high doses of PPIs throughout the duration of the Hp-EuReg. Low doses of PPIs were predominant in the beginning of the registry up until the year 2017; however, since the year 2017, after the release of updated Maastricht V/Florence consensus report, the rate of higher PPIs doses started increasing and is now predominantly represented by high-dose PPIs—almost half of the Hp-EuReg cases^39^.

Concerning *H. pylori* eradication regimens, we can state that the use of the main first and second-line prescriptions in the older and younger European populations met the recommendations of Maastricht V/Florence consensus report. However, the most frequently prescribed first-line therapy in both age groups, in spite of many countries with > 15% clarithromycin resistance rate, was standard triple therapy (PPI + C + A). Levofloxacin containing triple therapy was the most frequently used rescue regimen in both age groups, as previously recommended.

One of the main goals of this research was to evaluate the effectiveness of main first- and second-line *H. pylori* eradication regimens in the older and younger age groups. We have found that the overall first-line treatment effectiveness was very close to optimal (≥ 90%) eradication rates in younger subjects (89% by PP and 88% by mITT); whereas it was optimal by PP (90%) and very close to optimal by mITT (89%) in the older ones. The effectiveness of the most popular first-line prescription – standard triple therapy (PPI + C + A) was suboptimal in both age groups. Other triple therapies (PPI + C + M, PPI + A + L) did show even worse effectiveness. Optimal eradication rates were achieved only by using bismuth and non-bismuth-based quadruple therapies (PPI + C + A + B, PPI + C + A + T and single-capsule bismuth quadruple therapy (Pylera®)) and the most popular sequential therapy (PPI + C + A + T) in both age groups. The optimal effectiveness of these treatment regimens was also confirmed in other published studies^40–42^. Statistically significant differences in the effectiveness between the age groups were reported when standard triple therapy (PPI + C + A), quadruple PPI + C + A + M or sequential PPI + C + A + T therapies were used, while there were no differences in the remaining analysed prescriptions. In this respect, it is worthwhile mentioning that even though there were statistically significant differences between the age groups in various parameters, including the effectiveness of different prescriptions, these differences might be clinically non-significant and should be interpreted with caution, as most of them ranged between 1–2% and could be due merely to the very large sample size. Therefore, in most of the cases, we considered these differences to be clinically irrelevant, even though statistically significant.

The overall effectiveness of second-line treatment was suboptimal (84%) both by PP and mITT in both age groups. The effectiveness of the main second-line regimen (PPI + A + L) was suboptimal in both age groups. In fact, the only regimen that achieved optimal eradication rate in the older age group was the single-capsule bismuth quadruple therapy (Pylera^®^). In the younger group optimal eradication rates were achieved by using triple PPI + A + Mx and quadruple PPI + C + A + B prescriptions. In fact, the only statistically significant difference in second-line treatment effectiveness between the older and younger adults was obtained with the previously mentioned bismuth quadruple PPI + C + A + B prescription (80% vs. 91% by mITT respectively).

The multivariate analysis revealed the expected results – non-bismuth and, especially, bismuth-based quadruple therapies (the most frequent being PPI + C + A + B, single capsule Pylera® and PPI + C + A + M) were associated with better mITT cure rates in the first-line treatment as our effectiveness analysis revealed that most of these bismuth-containing regimens achieved optimal or close-to-optimal eradication rates. This was also confirmed in other studies^4,35,36,41,42^ and the current guidelines are shifting towards bismuth-based quadruple therapies as the main *H. pylori* treatment regimen^4^ recommendation given clarithromycin resistance rates are increasing worldwide^23,43,44^.

Another possible issue, which albeit was not confirmed in our study, was the possibility of worse treatment compliance in older subjects. Even though the older-aged populations were associated with a higher number of concurrent medications, the treatment compliance was very satisfactory in both age groups, reaching 97%.

Interestingly, the older adults experienced statistically significantly less AEs compared to the younger group; however, we should consider whether this difference is really clinically relevant and may be due, as previously stated, to the very large number of subjects in both age groups. Nonetheless, both age groups presented a similar safety profile (77% of the older and 75% of the younger adults without any AEs), whereas severe AEs were slightly more frequent in the older-aged subjects.

We can compare our study results to only a few other available similar studies. A Japanese study in the year 2019 also compared the diagnostics, efficacy and safety of *H. pylori* eradication between the age groups (younger (≤ 65 years), old (65–74 years), and super-old (≥ 75 years)). The study reported similar indications (chronic gastritis, PUD) for the eradication; however, the AEs rate in the old (9%) and super-old (12%) groups was significantly lower as compared to those in our study. Compared to our analysis, this study also reported a better effectiveness of the main standard triple therapy (PPI + A + C), which achieved optimal overall eradication rate (92%). When comparing the age groups, super-old patients had a significantly less frequent indication of chronic gastritis but more frequent indications of PUD compared to other groups. In this same Japanese study, the *H. pylori* eradication rates for older patients were not reported lower when compared to the younger patients. No remarkable differences were seen among the groups for the efficacy of prescribed regimens, no significant differences were observed in comparisons of AE rates among the groups^21^. Another small Chinese study also analysed the efficacy of *H. pylori* eradication between the age groups by using bismuth-based quadruple therapies for 14 days and did not yield significant differences either in the ITT and PP analysis between the age groups. This study also reported excellent eradication rates (> 92%) in both age groups^22^.

One of the main weaknesses of our study is the possible heterogeneity of the data. The Hp-EuReg currently includes 32 European countries and various regions might have different approaches to the management of *H. pylori* infection, which could be affected by diverse factors, such as the availability of local antimicrobial resistance rates, financial capabilities, availability of diagnostic methods, local antibiotics market as well as the knowledge and objectivity of the gastroenterologists. Our study has included a very large European cohort; providing accurate Pan-European data. Such is the case of previously published available studies from Hp-EuReg from different European countries^39^, where, despite the aforementioned concerns, it has to be acknowledged that multicentre collaboration gathering information on the daily routine of the gastroenterological practice is one of the best ways to secure a critical mass of knowledge encompassing the inclusion of those even difficult-to-treat cases as well as offering power to the statistical analyses.

## Conclusions

In general, the approach to the diagnostics and treatment of *H. pylori* infection did not differ between the older and younger European populations. The main differences were reported in the concurrent medications, allergy to penicillin, AEs and the type of prescribed regimen (triple vs. quadruple) both in first- and second-line treatment, which were generally well-tolerated in both age groups. The effectiveness of the most frequent first- and second-line triple therapies in the younger and older populations was suboptimal (< 90%) and optimal effectiveness rates (> 90%) were mostly achieved by using bismuth and non-bismuth-based quadruple therapies (quadruple PPI + C + A + M, quadruple PPI + C + A + B, quadruple PPI + C + A + T, single capsule bismuth quadruple Pylera®, sequential C + A + T for the first line first-line treatment; quadruple PPI + C + A + B (only in the younger adults), single capsule bismuth Pylera® for the second-line treatment). Although statistically significant, no clinically relevant differences in the effectiveness between older and younger patients was observed in the most frequently prescribed first- and second-line prescriptions.

### Supplementary Information

Supplementary Information.

## Data Availability

*Data transparency statement*: Raw data were generated at AEG-REDCap. Derived data supporting the findings of this study are available from the Hp-EuReg Scientific Director and the PI of the project (OPN and JPG) upon request. *Data sharing statement:* The data that support the findings of this study are not publicly available given that containing information could compromise the privacy of research participants. However, previous published data on the Hp-EuReg study, or de-identified raw data referring to current study, as well as further information on the methods used to explore the data could be shared, with no particular time constraint. Individual participant data will not be shared.
