# Author Correction: Comparison of the management of *Helicobacter pylori* infection between the older and younger European populations

**DOI:** 10.1038/s41598-023-45888-5

**Published:** 2023-11-01

**Authors:** Paulius Jonaitis, Olga P. Nyssen, Ilaria Maria Saracino, Giulia Fiorini, Dino Vaira, Ángeles Pérez-Aísa, Bojan Tepes, Manuel Castro-Fernandez, Manuel Pabón-Carrasco, Alma Keco-Huerga, Irina Voynovan, Alfredo J. Lucendo, Ángel Lanas, Samuel J. Martínez-Domínguez, Enrique Alfaro Almajano, Luis Rodrigo, Ludmila Vologzanina, Natasa Brglez Jurecic, Maja Denkovski, Luis Bujanda, Umud Mahmudov, Mārcis Leja, Frode Lerang, Gülüstan Babayeva, Dmitry S. Bordin, Antonio Gasbarrini, Juozas Kupcinskas, Oleksiy Gridnyev, Theodore Rokkas, Ricardo Marcos-Pinto, Perminder S. Phull, Sinead M. Smith, Ante Tonkić, Doron Boltin, György Miklós Buzás, Štěpán Šembera, Halis Şimşek, Tamara Matysiak-Budnik, Vladimir Milivojevic, Wojciech Marlicz, Marino Venerito, Lyudmila Boyanova, Michael Doulberis, Lisette G. Capelle, Anna Cano-Català, Leticia Moreira, Francis Mégraud, Colm O’Morain, Javier P. Gisbert, Laimas Jonaitis, Hp-EuReg investigators, Renāte Būmane, Emin Mammadov, Rustam A. Abdulkhakov, Galina Fadeenko, Jose M. Huguet

**Affiliations:** 1 Department of Gastroenterology, Lithuanian University of Health Sciences, 50161 Kaunas, Lithuania; 2 Gastroenterology Unit, Hospital Universitario de La Princesa, Instituto de Investigación Sanitaria Princesa (IIS-Princesa), Centro de Investigación Biomédica en Red de Enfermedades Hepáticas y Digestivas (CIBERehd), Universidad Autónoma de Madrid (UAM), Diego de León, 62, 28006 Madrid, Spain; 3 Department of Surgical and Medical Sciences, IRCCS AOUBO, University of Bologna, 40138 Bologna, Italy; 4 Agencia Sanitaria Costa del Sol, Red de Investigación en Servicios de Salud en Enfermedades Crónicas (REDISSEC), 29651 Marbella, Spain; 5 Department of Gastroenterology, AM DC Rogaska, 3250 Rogaska Slatina, Slovenia; 6 Department of Gastroenterology, Hospital de Valme, 41014 Seville, Spain; 7 Department of Gastroenterology, A.S. Loginov Moscow Clinical Scientific Center, 111123 Moscow, Russia; 8 Department of Gastroenterology, Hospital General de Tomelloso, 13700 Tomelloso, Spain; 9 IIS Aragón y Facultad de Medicina de la Universidad de Zaragoza, 50009 Zaragoza, Spain; 10 Gastroenterology Unit, Hospital Universitario Central de Asturias, 33011 Oviedo, Spain; 11 Department of Gastroenterology, Gastrocentre, 614068 Perm, Russia; 12 Department of Gastroenterology, Interni Oddelek, Diagnostic Centre, 4260 Bled, Slovenia; 13 Hospital Donostia, Instituto Biodonostia, Centro de Investigación Biomédica en Red de Enfermedades Hepáticas y Digestivas (CIBERehd), Universidad del País Vasco (UPV/EHU), 20018 San Sebastián, Spain; 14 Modern Hospital, 1119 Baku, Azerbaijan; 15 Department of Gastroenterology, Digestive Diseases Centre Gastro, Institute of Clinical and Preventive Medicine and Faculty of Medicine, University of Latvia, Riga, 1079 Latvia; 16 Department of Gastroenterology, Østfold Hospital Trust, 1714 Grålum, Norway; 17 Memorial Klinik, 1096 Baku, Azerbaijan; 18 Department of Gastroenterology, A.S. Loginov Moscow Clinical Scientific Center, 111123 Moscow, Russia; 19 A.I. Yevdokimov Moscow State University of Medicine and Dentistry, 127473 Moscow, Russia; 20 Tver State Medical University, 170100 Tver, Russia; 21 Medicina Interna, Fondazione Policlinico Universitario A. Gemelli Istituto di Ricovero e Cura a Carattere Scientifico, Università Cattolica del Sacro Cuore, 00168 Rome, Italy; 22 Government Institution “L.T.Malaya Therapy National Institute of the National Academy of Medical Sciences of Ukraine”, Kyiv, Ukraine; 23 Department of Gastroenterology, Henry Dunant Hospital, 115 26 Athens, Greece; 24 Department of Gastroenterology, Centro Hospitalar do Porto Institute of Biomedical Sciences Abel Salazar, Centro de Investigação em Tecnologias e Serviços de Saúde, University of Porto, 4050-313 Porto, Portugal; 25 Department of Gastroenterology, Aberdeen Royal Infirmary, Aberdeen, AB25 2ZN UK; 26 Faculty of Health Sciences, Trinity College Dublin, Dublin, D02PN40 Ireland; 27 Department of Gastroenterology, University Hospital of Split, University of Split School of Medicine, 21000 Split, Croatia; 28 Division of Gastroenterology, Rabin Medical Center, Sackler School of Medicine, Tel Aviv University, 49100 Tel Aviv, Israel; 29 Department of Gastroenterology, Ferencváros Health Centre, 1095 Budapest, Hungary; 30 2nd Department of Internal Medicine and Gastroenterology, University Hospital and Charles University, Faculty of Medicine in Hradec Králové, 500 03 Hradec Králové, Czech Republic; 31 Division of Gastroenterology and Hepatology, Hacettepe University School of Medicine, 06230 Ankara, Turkey; 32 Department of Gastroenterology, CHRU de Nantes, Hôpital Hôtel Dieu, 44000 Nantes, France; 33 Department of Gastroenterology, Clinical Center of Serbia, University of Belgrade School of Medicine, 11000 Belgrade, Serbia; 34 Department of Gastroenterology, Pomeranian Medical University, 70-204 Szczecin, Poland; 35 Department of Gastroenterology, Otto-Von-Guericke University, 39120 Magdeburg, Germany; 36 Department of Gastroenterology, Medical Microbiology, Medical University of Sofia, 1431 Sofia, Bulgaria; 37 Division of Gastroenterology and Hepatology, Medical University Department, Kantonsspital Aarau, 5001 Aarau, Switzerland; 38 Department of Gastroenterology, Meander Medical Center, 3813 TZ Amersfoort, The Netherlands; 39 GOES Research Group, Althaia Xarxa Assistencial Universitària de Manresa, 08243 Manresa, Spain; 40 Department of Gastroenterology, Hospital Clínic Barcelona, Centro de Investigación Biomédica en Red en Enfermedades Hepáticas y Digestivas (CIBEREHD), IDIBAPS (Institut d’Investigacions Biomèdiques August Pi i Sunyer), University of Barcelona, 08036 Barcelona, Spain; 41 INSERM U1312, Université de Bordeaux, 33000 Bordeaux, France; 42 Internal Medicine and Gastroenterology Department, Azerbaijan State Advanced Training Institute for Doctors named after A. Aliyev, Baku, Azerbaijan; 43 Kazan State Medical University, Kazan, Tatarstan, Russia; 44 Digestive Ukrainian Academy of Medical Sciences, Kyiv, Ukraine; 45 Gastroenterology Unit, Consorci Hospital General Universitari Valencia, Valencia, Spain

Correction to: *Scientific Reports* https://doi.org/10.1038/s41598-023-43287-4, Published online 11 October 2023

In the original version of this Article, ‘Hp-EuReg investigators’ was omitted from the author list. The details regarding the Hp-EuReg investigators were provided in the Supplementary Information.

The original Article has been corrected.

