# Supplementary material for: Comparison of the management of Helicobacter pylori infection between the older and younger European populations
Source: Sci Rep. 2023 Oct 11;13:17235. doi: 10.1038/s41598-023-43287-4 (PMC10567783; doi:10.1038/s41598-023-43287-4)
Supplement: Supplementary file 1 — Supplementary Information. [file 41598_2023_43287_MOESM1_ESM.docx]

Supplementary file 1. **Hp-EuReg investigators**

Renāte Būmane, Department of Gastroenterology, Digestive Diseases Centre Gastro, Institute of Clinical and Preventive Medicine and Faculty of Medicine, University of Latvia, Riga, LATVIA Acquired data, critically reviewed the manuscript draft, and approved the submitted manuscript.

Emin Mammadov, Internal medicine and gastroenterology department, Azerbaijan State Advanced Training Institute for Doctors named after A. Aliyev, Baku, AZERBAIJAN Acquired data, critically reviewed the manuscript draft, and approved the submitted manuscript.

Rustam A Abdulkhakov, Kazan State Medical University, Kazan, Tatarstan, RUSSIA Acquired data, critically reviewed the manuscript draft, and approved the submitted manuscript.

Galina Fadeenko, Digestive Ukrainian Academy of Medical Sciences, Kyiv, UKRAINE Acquired data, critically reviewed the manuscript draft, and approved the submitted manuscript.

Jose M. Huguet, Gastroenterology Unit, Consorci Hospital General Universitari Valencia, Valencia, SPAIN Acquired data, critically reviewed the manuscript draft, and approved the submitted manuscript.
